# Editorial: Innovative field diagnostics for real-time plant pathogen detection and management

**DOI:** 10.3389/fpls.2026.1877895

**Published:** 2026-05-19

**Authors:** Ravinder Kumar, Milan Kumar Lal, Rahul Kumar Tiwari, Anirban Roy, A. Abdul Kader Jailani, M. N. Maruthi

**Affiliations:** 1 Division of Plant Pathology, Advanced Centre for Plant Virology, ICAR-Indian Agricultural Research Institute, New Delhi, India; 2 Division of Crop Physiology & Biochemistry, ICAR-National Rice Research Institute, Cuttack, Odisha, India; 3 Division of Crop Protection, ICAR-Indian Sugarcane Research Institute, Lucknow, India; 4 Plant Pathology Department, North Florida Research and Education Center, University of Florida, Quincy, FL, United States; 5 Agriculture, Health and Environment Department, Natural Resources Institute, University of Greenwich, Chatham, Kent, United Kingdom

**Keywords:** artificial intelligence, field level diagnostics, molecular diagnostics, non-invasive diagnostics, plant pathogen

Plant diseases are estimated to cause 20-40% of global crop losses annually, and their effective control depends critically on timely and accurate diagnosis (Shafay et al., 2025). However, conventional diagnostic approaches, including visual inspection, culture-based identification, and laboratory-dependent molecular assays, often fall short in providing rapid and actionable insights, particularly at early stages of infection (Kumar et al., 2023; Paul et al., 2025). These limitations emphasize the urgent need for innovative, field-deployable diagnostic technologies that enable real-time detection and informed decision-making. This Research Topic addresses this need by bringing together fourteen high-quality contributions that collectively advance the science and practice of plant disease diagnostics. These studies span a wide spectrum of disciplines, including molecular biology, spectroscopy, artificial intelligence (AI), epidemiology, and agroecosystem management.

## Advancing molecular diagnostics: precision, sensitivity, and predictive capability

Molecular diagnostics remain a cornerstone of plant pathogen detection, and several contributions in this Research Topic demonstrate significant advancements in sensitivity, specificity, and applicability under field conditions. Traditional PCR-based methods have evolved into more sophisticated platforms capable of multiplex detection and quantitative analysis, enabling not only pathogen identification but also assessment of disease risk. Singh et al. present a notable advancement through the development of a multipurpose diagnostic marker targeting a bromodomain-containing gene unique to *Rhizoctonia solani* AG1-IA. By integrating conventional PCR, quantitative PCR (qPCR), and loop-mediated isothermal amplification (LAMP), the study demonstrates robust detection across plant tissues and soil matrices. The ability to detect pathogens directly in soil is particularly important for managing soil-borne diseases, where early detection can significantly influence crop management and resistance breeding strategies. Similarly, Liu et al. developed a multiplex TaqMan real-time PCR assay for simultaneous detection of three major *Fusarium* species causing root rot in red kidney bean. Beyond detection, their study established a strong quantitative relationship between pathogen DNA copy number and disease severity, enabling predictive modeling of disease progression. This integration of molecular diagnostics with statistical modeling represents a critical step toward actionable disease forecasting and targeted interventions.

## Spectroscopy and non-invasive diagnostics: a new frontier

Next-generation diagnostics increasingly rely on non-destructive, real-time monitoring. Spectroscopy-based methods, particularly Raman spectroscopy and hyperspectral imaging, have emerged as powerful tools for detecting subtle biochemical and physiological changes associated with pathogen infection. Kuo et al. demonstrated the potential of Raman spectroscopy for early detection of fungal infections in *Arabidopsis thaliana* and *Brassica* species. Their study revealed that Raman spectral signatures could detect infection-induced changes before visible symptoms appeared, providing a critical window for early intervention. The identification of infection response indices further highlights the capacity of this technique to quantify plant responses to pathogen attack. Wang et al. extended this approach by integrating Raman spectroscopy with advanced machine learning models to detect and classify *Verticillium* wilt severity in cotton stems. The study achieved high classification accuracy, demonstrating the synergy between spectral data and AI algorithms. Importantly, this approach enables not only detection but also severity assessment, which is crucial for guiding management decisions. In parallel, Gao et al. utilized hyperspectral imaging combined with machine learning to detect *Verticillium* wilt in cotton leaves. Their findings revealed that disease signals are more pronounced in lower canopy leaves during early infection stages, emphasizing the importance of spatial resolution and plant architecture in disease detection. Further advancing imaging-based diagnostics, Wei et al. addressed the challenge of precise disease grading in walnut leaf brown spot. Their proposed CogFuse-MobileViT model integrates hierarchical feature selection and adaptive multi-scale dilated convolution fusion to improve detection of blurred lesion edges and complex features. The model significantly outperformed baseline architectures, demonstrating the importance of feature optimization in disease severity assessment. Wang et al. provided a broader perspective on optical spectroscopy technologies, highlighting the role of both labeled and label-free approaches. Micro-Raman spectroscopy, in particular, enables rapid, non-invasive discrimination of viable but non-culturable pathogens at the single-cell level. However, the authors also emphasize current limitations, including the need for comprehensive spectral databases and challenges in detecting previously unknown pathogens.

## Artificial intelligence and deep learning: enabling automation and scalability

Artificial intelligence has rapidly become a central component of modern plant disease diagnostics, offering exceptional capabilities for image analysis, pattern recognition, and decision support. Several contributions in this Research Topic highlight the application of advanced AI models to plant disease detection and management. Aldhyani and Alkahtani developed transformer-based models, including Vision Transformers, for detecting palm leaf diseases and Dubas insect infestations. Their models achieved remarkable accuracy, demonstrating the effectiveness of attention-based architectures in capturing complex visual features. Liu et al. employed advanced YOLO-based object detection models for real-time disease identification in greenhouse crops. Their approach addresses practical challenges such as occlusion, variable lighting, and complex backgrounds, making it highly relevant for real-world applications. Iqbal introduced a comprehensive AI framework that integrates classification, detection, segmentation, and severity estimation using vision-language models and generative AI. This unified approach represents a significant advancement over traditional methods, which typically treat these tasks independently. Despite these advances, high computational demand still limits field-scale deployment, particularly in resource-constrained settings. Natij et al. addressed this challenge by proposing a resource-efficient framework that integrates reduced-order modeling with classical machine learning. Their approach significantly reduces computational complexity while maintaining high classification accuracy, enabling deployment on low-resource devices. Together, these contributions highlight the transition toward automated, scalable, and resource-efficient diagnostic systems, capable of operating across diverse agricultural settings.

## Integrating diagnostics with epidemiology and climate dynamics

Plant disease development is inherently influenced by environmental conditions, and effective management requires integration of diagnostic tools with epidemiological and climatic data. Several studies in this Research Topic emphasize this systems-level perspective. Sharma et al. developed predictive models for *Sclerotinia* stem rot in *Brassica juncea* based on long-term meteorological data. Their findings demonstrate how temperature, humidity, and sunshine hours’ influence disease dynamics, enabling accurate forecasting of disease risk and identification of optimal sowing windows. Yele et al. examined the impact of climate change variables, including elevated CO_2_, temperature, and ozone, on pest populations and crop productivity. Their study highlights the need for diagnostic systems that can adapt to changing environmental conditions. Asinari et al. explored the influence of soil management practices on airborne fungal communities and disease pressure in vineyards. Their findings indicate that soil tillage and mulching practices can modulate microbial dynamics, although their effects are strongly influenced by climatic conditions. These studies underscore the importance of integrating diagnostics with environmental monitoring and predictive modeling, enabling proactive and sustainable disease management strategies.

## Challenges and future directions

Despite the significant progress highlighted in this Research Topic, several challenges remain. Translating advanced diagnostic technologies into practical field applications requires robust validation across diverse crops, environments, and pathogen systems. Issues related to data standardization, interoperability, and model generalization must be addressed to ensure reliability and scalability. Moreover, widespread adoption depends on affordability, accessibility, and user-friendly interfaces. Bridging the gap between research innovation and field implementation will require strong interdisciplinary collaboration among plant scientists, engineers, data scientists, policymakers, and farmers. Future research should prioritize the development of integrated diagnostic platforms that combine sensing, analytics, and decision support. Advances in genomics, microbiome analysis, and remote sensing will further enhance predictive capabilities and enable precision agriculture at unprecedented scales.

## Conclusion

The fourteen contributions in this Research Topic collectively represent a significant advancement in plant disease diagnostics and management. By integrating molecular biology, spectroscopy, artificial intelligence, and agroecological insights, these studies lay the foundation for a new generation of real-time, field-deployable plant health monitoring systems. These innovations hold immense promise for improving disease detection, enhancing management strategies, and reducing crop losses. As global agricultural systems face increasing challenges, the development and adoption of such integrated diagnostic approaches will be essential for ensuring food security and sustainability. We hope this Research Topic catalyses interdisciplinary collaboration among plant pathologists, data scientists, and engineers, accelerating the translation of these innovations from laboratory to field, and from individual diagnostics to integrated decision-support platforms.
